# Nonsynonymous variants in the *SMAD6* gene predispose to congenital cardiovascular malformation

**DOI:** 10.1002/humu.22030

**Published:** 2012-01-20

**Authors:** Huay L Tan, Elise Glen, Ana Töpf, Darroch Hall, John J O'Sullivan, Linda Sneddon, Christopher Wren, Peter Avery, Richard J Lewis, Peter ten Dijke, Helen M Arthur, Judith A Goodship, Bernard D Keavney

**Affiliations:** 1 Institute of Genetic Medicine, Newcastle UniversityNewcastle upon Tyne, UK; 2 Newcastle upon Tyne Hospitals NHS Foundation TrustNewcastle upon Tyne, UK; 3 School of Mathematics & Statistics, Newcastle UniversityNewcastle upon Tyne, UK; 4 Institute for Cell and Molecular Biosciences, Newcastle UniversityNewcastle upon Tyne, UK; 5 Department of Molecular Cell Biology and Centre for Biomedical Genetics, Leiden University Medical CenterLeiden, the Netherlands

**Keywords:** rare genetic variants, congenital heart defects, BMP, SMAD

## Abstract

Congenital cardiovascular malformation (CVM) exhibits familial predisposition, but most of the specific genetic factors involved are unknown. Postulating that rare variants in genes in critical cardiac developmental pathways predispose to CVM, we systematically surveyed three genes of the bone morphogenetic protein (BMP) signaling pathway for novel variants. Exonic, splice site, and untranslated regions of *BMPR1A*, *BMPR2*, and *SMAD6* genes were sequenced in 90 unrelated sporadic cases of CVM. One nonsynonymous variant (p.C484F) with predicted functional impact was found in the MAD homology 2 domain of *SMAD6*, an intracellular inhibitor of BMP signaling. Sequencing this domain in an additional 346 cases of CVM yielded two further nonsynonymous variants (p.P415L and p.A325T). Functional effects of all three SMAD6 mutations were investigated using BMP signaling assays in vitro. Two SMAD6 variants (p.C484F and p.P415L) had significantly (*P* < 0.05) lower activity than wild-type SMAD6 in inhibiting BMP signaling in a transcriptional reporter assay. In addition, the p.C484F variant had a significantly (*P* < 0.05) lower capacity to inhibit an osteogenic response to BMP signaling. We conclude that low-frequency deleterious variants in *SMAD6* predispose to CVM. This is the first report of a human disease phenotype related to genetic variation in *SMAD6.* Hum Mutat 33:720–727, 2012. © 2012 Wiley Periodicals, Inc.

## Introduction

Cardiovascular malformation (CVM) affects around seven in 1,000 live births and is a major cause of mortality and morbidity in childhood in the western countries. In 80% of “sporadic” cases that cannot be attributed to particular malformation syndromes or teratogenic exposures, there remains a substantial excess familial risk, indicating a significant genetic contribution to disease susceptibility [Burn et al., 1998; Oyen et al., 2009]. These recurrence risks are compatible with the presence of multiple genetic risk variants of incomplete penetrance, likely interacting with environmental factors. The majority of genetic factors predisposing to non-Mendelian CVM remain to be identified. We therefore postulated that rare variants in genes that regulate cardiac development may predispose individuals to CVM.

**Figure 1:**
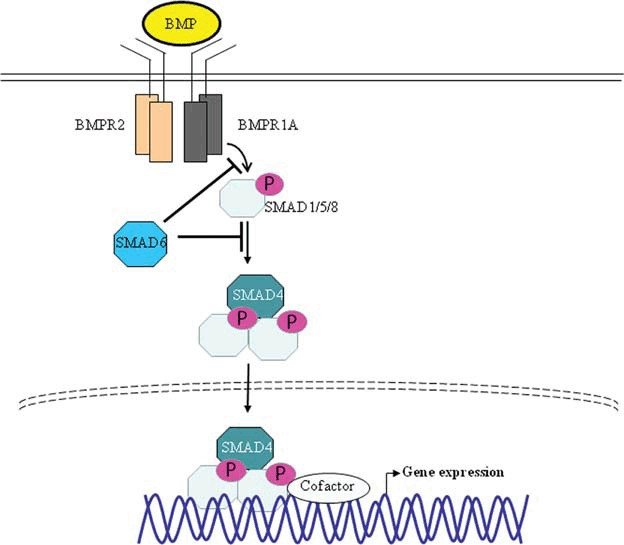
Diagrammatic representation of BMP signaling pathway. Bone morphogenetic proteins (BMPs) are members of the transforming growth factor β superfamily of cytokines and signal through serine/threonine receptors on the cell surface. BMP ligands bind to the BMP type I receptor BMPR1A or BMPR1B (also known as ALK6; not shown) in complex with the BMP type II receptor (BMPR2). Binding of ligand to the receptor complex stimulates BMPR1A to phosphorylate SMAD1/5/8 transcription factors, which translocate to the nucleus in combination with SMAD4 protein to regulate BMP-responsive genes. SMAD6 inhibits BMP signaling by inhibiting BMPR1A and BMPR1B activity [Goto et al., 2007; Imamura et al., 1997] or by competing with SMAD4 for binding with phosphorylated SMAD1 [Hata et al., 1998], as indicated in the figure. It can also inhibit BMP signaling by recruiting Smad ubiquitin regulatory factor to activated BMP receptors to prime them for degradation [Murakami et al., 2003] and by binding certain transcription factors to repress transcription in the nucleus [Bai et al., 2000].

Members of the bone morphogenetic protein (BMP) signaling pathway (Fig. 1) are known to be important regulators of cardiogenesis [Wang et al., 2011]. BMP signaling is required for normal heart valve and outflow tract development [Goldman et al., 2009; Jiao et al., 2003; Liu et al., 2004; Ma et al., 2005; Rivera-Feliciano and Tabin, 2006; Sugi et al., 2004; Zhang and Bradley, 1996]. BMP ligands regulate cell responses by interacting with specific serine/threonine kinase receptors on the cell surface, as summarized in Figure 1. Of the BMP signaling receptors, BMP receptor type1A (BMPR1A, also known as ALK3) is required in endothelial cells for normal development of the cardiac cushions [Kaneko et al., 2008; Song et al., 2007], in myocardial cells for normal trabeculae and endocardial cushion formation [Gaussin et al., 2005], and in neural crest for normal outflow tract septation [Stottmann et al., 2004]. BMPR2 is required for normal development of the heart valves, septa, and outflow tract [Beppu et al., 2009; Delot et al., 2003]. The level of BMP signaling is regulated by the inhibitory protein SMAD6, which is highly expressed in the cardiac valves and outflow tract of the embryonic heart and is upregulated by shear stress [Galvin et al., 2000; Topper et al., 1997]. SMAD6 inhibits BMP signaling and this negative regulation is also important during heart development, as *Smad6* knockout mice have cardiac valve defects and aortic ossification [Galvin et al., 2000].

As correct functioning of the BMP signaling pathway is required for normal heart development, we hypothesized that rare functional variation in members of this pathway could predispose individuals to increased risk of CVMs. We therefore screened patients with a range of CVM phenotypes for nonsynonymous mutations in the coding region of *BMPR2* (MIM# 600799), *BMPR1A* (MIM# 601299), and *SMAD6* (MIM# 602931) genes. We identified three nonsynonymous *SMAD6* mutations, which were further investigated in laboratory assays for their effects on the ability of SMAD6 protein to regulate BMP signaling.

## Materials and Methods

### Sample Collection

Caucasian patients of British ancestry affected by CVM were recruited at the Newcastle upon Tyne Hospitals NHS Foundation Trust, Newcastle, UK. Ethics approval was granted by the Northumberland Research Ethics Committee. Fully informed written consent was obtained from all participants or their parents. Patients with chromosomal abnormalities, other multiorgan malformation syndromes, learning difficulties, known maternal exposure to significant teratogens during pregnancy, or family histories suggestive of Mendelian inheritance were excluded. DNA was extracted from peripheral blood or saliva using standard procedures.

### Resequencing/Mutation Detection

The entire coding sequences, including exon–intron boundaries, of *BMPR2* (NM_001204.6), *BMPR1A* (NM_004329.2), and *SMAD6* (NM_005585.4) were resequenced in 90 CVM probands. Binomial probability indicates that if mutations in the screened sequence were present in 2.5% or greater of CVM cases, sequencing this number of probands would have 80% power of detecting at least one such mutation. Bidirectional sequencing was performed using MegaBACE 1000 sequencer (GE Healthcare). The MAD homology 2 (MH2) domain of *SMAD6* encoded by exon 4 was resequenced in an additional 346 probands. Previously unreported nonsynonymous variants identified in the CVM patients were genotyped in a collection of 1,000 Caucasian controls of British ancestry free of CVM [Palomino-Doza et al., 2008], using iPLEX (Sequenom, Hamburg, Germany) or custom Taqman (Applied Biosystems, Paisley, UK). To estimate the frequency of nonsynonymous variants in the MH2 domain of SMAD6 in healthy controls, SMAD6 sequence data from 629 individuals available from the 1000 Genomes Project (http://browser.1000genomes.org/index.html) and from 200 exomes of Danish individuals (http://soap.genomics.org.cn/) were analyzed for all nucleotide changes [Durbin et al., 2010; Li et al., 2010].

### Cell Culture and Transfections

Mouse myoblast C2C12 cells (American Type Culture Collection, LGC Standards, Teddington, UK) were maintained in Dulbecco's modified Eagle's medium in 10% fetal bovine serum with 50 units/ml penicillin and 50 μl/ml streptomycin (Invitrogen, Paisley, UK) at 37°C in 5% CO_2_-humidified atmosphere. Cells were transfected using FuGENE HD transfection reagent (Roche Diagnostics) according to the manufacturer's recommendations.

### Constructs

Plasmids expressing the MH2 domain, corresponding to the C-terminal region of SMAD6 protein (SMAD6C), and a constitutively active BMPR1A receptor (caBMPR1A) have been previously described [Fujii et al., 1999; Hanyu et al., 2001]. *SMAD6* mutations c.1244C>T (p.P415L), c.973G>A (p.A325T), and c.1451G>T (p.C484F) were introduced into the *SMAD6C* expression construct by site-directed mutagenesis using QuickChange kit (Stratagene-Agilent Technologies, Stockport, UK).

### Luciferase Assay

Dual luciferase assays were performed using previously validated luciferase transcriptional reporter constructs [Goto et al., 2007] containing BMP/SMAD responsive elements (BRE–luc) [Korchynskyi and ten Dijke, 2001]. C2C12 cells were seeded in 12-well plates for 24 hr before being transfected with caBMPR1A; BRE–luc; and either wild-type (wt) SMAD6C, mutant SMAD6C constructs, or empty vector pcDNA3.1 (Invitrogen). A pRL–TK vector (in which *Renilla luciferase* is driven by a thymidine kinase promoter) was cotransfected in all samples as a control for transfection efficiency. Cells were incubated for 24 hr after transfection and luciferase activities were measured in lysates using Dual-Luciferase Reporter Assay System (Promega, Southampton, UK) following manufacturer's protocol. Data were normalized using *Renilla* luciferase activity. Three independent experiments were performed (each in triplicate).

### Immunoblotting

After reading the luciferase activity, NuPAGE sample reducing agent and sample buffer (Invitrogen) were added to aliquots of cell lysates. Proteins were separated by sodium dodecyl sulfate-polyacrylamide gel electrophoresis and transferred to Hybond-C extra nitrocellulose membrane (GE Healthcare, Little Chalfont, UK). Antibodies used were anti-FLAG M2-peroxidase (1:10,000; Sigma, Poole, UK), anti-heme agglutinin-peroxidase 3F10 (1:1,000; Roche Diagnostics, Burgess Hill, UK), anti-α-tubulin DM1A (1:10,000; Sigma), and anti-nucleolin (1:10,000; Bethyl Laboratories, Montgomery, Texas). An enhanced chemiluminescent detection system (Thermo Fisher Scientific, Basingstoke, UK) was used to detect the antibodies.

### Alkaline Phosphatase Assay

Alkaline phosphatase (ALP) activity was measured in C2C12 cells transfected with *SMAD6* variants. Cells were transfected with caBMPR1A and either wt SMAD6C, mutant SMAD6C constructs, or empty vector pcDNA3.1, and were cultured for 3 days with one change of medium. Cells were then washed with phosphate-buffered saline, lysed with 50 mM Tris-HCl (pH 7.5) and 0.1% Triton X-100 and sonicated. ALP activity was determined using *p*-nitrophenyl phosphate in 100 mM glycine, 1 mM MgCl_2_, and 0.1 mM ZnCl_2_ (pH 10.5), as previously described [Katagiri et al., 1994]. Protein concentration of cell lysates was measured using a BCA kit (Thermo Scientific).

### Homology Modeling

In the absence of a structure of SMAD6, a homology model was generated based on 34% sequence identity to the MH2 domain of SMAD1, the crystal structure of which is available (Protein Data Bank [PDB] ID, 1KHU). The sequence alignment between SMAD1 and SMAD6 was generated using the default settings of ClustalW2 (www.ebi.ac.uk/Tools/clustalw2/index.html) and was used as the template to thread the SMAD6 amino acid sequence onto the SMAD1 structure using CCP4 module Chainsaw [Stein, 2008]. Manual correction of the model was performed in Coot [Emsley and Cowtan, 2004] and was then conjugate gradient energy minimized for 200 cycles with crystallography and NMR system [Brunger et al., 1998]. To create the p.C484F mutant model, the cysteine at position 484 (Cys484) was replaced by the most commonly observed phenylalanine (Phe) rotamer in the Coot library and energy minimized as mentioned above. Figures were produced using the PyMOL Molecular Graphics System (Schrödinger, Camberley, UK).

### Statistical Analysis

Results are expressed as mean ± SEM. Data for the luciferase assay were logarithmic transformed to achieve approximate normality. No transformation was needed for the ALP assay. Statistical significance was determined by two-way analysis of variance and subsequent pairwise comparisons were performed, all using Minitab 15 Statistical Software (Minitab Ltd., Coventry, UK).

## Results

### Identification of Nonsynonymous Coding Region Mutations in Patients with CVM

The cardiac phenotypes of the patients are described in Supp. Table S1. We adopted a two-stage strategy consisting of a “discovery cohort” of 90 patients and a “replication cohort” of 346 patients. The first group of 90 patients was screened for novel sequence variants in the coding region of *BMPR2*, *BMPR1A*, and *SMAD6* genes. A nonsynonymous variant in exon 4 of *SMAD6*, which corresponded to a cysteine to phenylalanine substitution at position 484 of the protein (p.C484F), was found in a patient with bicuspid aortic valve, aortic valvar stenosis, and coarctation and calcification of the aorta (Supp. Table S1). No nonsynonymous variants were found in the coding region of *BMPR1A* or *BMPR2*. The p.C484F variant identified in SMAD6 was absent from the 1,000 Caucasian controls of British ancestry free of CVM [Palomino-Doza et al., 2008].

**Figure 2:**
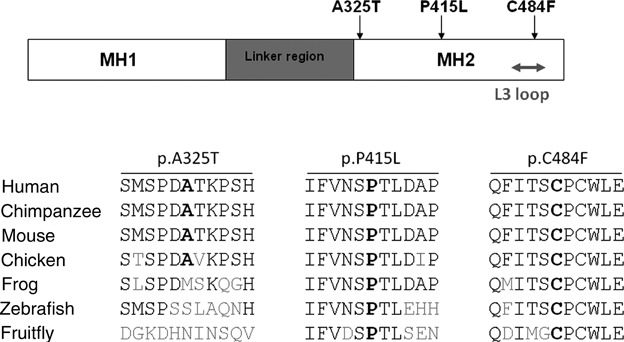
Location of mutations in SMAD6 protein. Diagram to illustrate the position of the three mutations in the MAD homology (MH)2 domain (encoded by exon 4) at the carboxy terminus of SMAD6. This is separated from the MH1 domain at the N-terminus by a linker region. Mutation p.C484F lies within the L3 loop region, which interacts with the BMP type I receptor. Protein alignments show the degree of evolutionary conservation of the mutated amino acid residues 325, 415, and 484 (in bold text).

The p.C484F mutation lies in the MH2 domain of *SMAD6* (Fig. 2), the function of which is critical for the protein's interaction with other members of the BMP signaling pathway. Resequencing of the MH2 domain of *SMAD6* (encoded by exon 4) in an additional 346 probands with a broad range of CVM phenotypes was therefore undertaken. This yielded two further nonsynonymous variants: an alanine to threonine substitution at position 325 of the protein (p.A325T) in a patient with congenital mitral regurgitation and a proline to leucine substitution at position 415 of the protein (p.P415L) in an infant with a bicuspid aortic valve and moderate aortic stenosis (Supp. Table S1). These three SMAD6 variants were not found in the publicly available single-nucleotide polymorphism (SNP) databases (dbSNP; www.ncbi.nlm.nih.gov/projects/SNP/). We next analyzed available sequencing data for the MH2 coding region of SMAD6 from 629 individuals in the 1000 Genomes Project and from 200 individuals in the Danish Exome Project [Durbin et al., 2010; Li et al., 2010]. There were no nonsynonymous variants. Thus, nonsynonymous variants in the MH2 domain of *SMAD6* were significantly more common in patients (3/436) than in controls (0/829; Yates' chi-squared = 3.152; degree of freedom = 1; one-tailed *P* value = 0.0375).

### Predicted Structural Changes in *SMAD6* Variants

**Figure 3:**
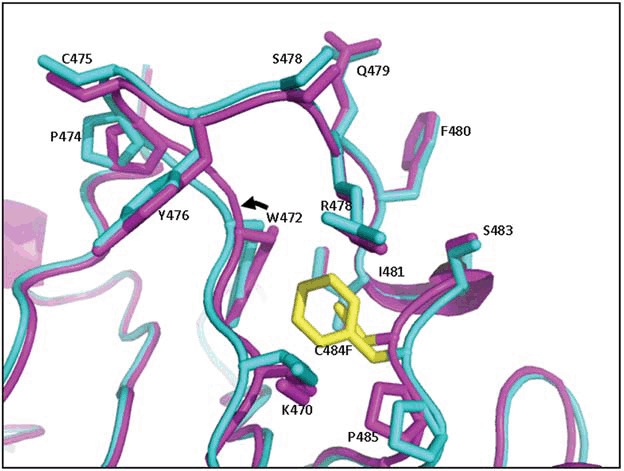
Model showing structural changes in the SMAD6 p.C484F variant. Homology models of wild-type (pink) and C484F (cyan) SMAD6 proteins are superimposed, with residue 484 highlighted in both models in yellow. Individual amino acids in the L3 loop are drawn as stick models and the remainder of the structure as a ribbon cartoon. Note that Trp472 in particular has to move (arrow) to accommodate the extra bulk presented by mutation of Cys484 to Phe.

Following the identification of three *SMAD6* variants within the MH2 domain (Fig. 2), the crystal structure of the SMAD1 homotrimeric complex (PDB entry, 1KHU), which is homologous to the MH2 domain of SMAD6, was utilized as a basis for modeling the structure of wt and variant SMAD6 MH2 domains. The change of cysteine to phenylalanine in the p.C484F variant resulted in significant changes in the structure that was limited to the immediate vicinity of residue 484. In the wt model, Cys484 is buried from solvent by the “L3 loop” of the MH2 domain and the thiol packs, in particular, against the indole ring of Trp472. To accommodate the extra bulk of a phenylalanine side chain, the structure in the immediate vicinity of the L3 loop adopts an altered conformation (Fig. 3). Glycines 471 and 473, which immediately flank the tryptophan, provide the potential for necessary flexibility in this part of the structure of the L3 loop during receptor binding, and the p.C484F variant may therefore affect receptor binding.

Similar comparative structural modeling of the p.P415L variant suggests that the α-helix H2, which, unusually, is N-capped by P415, is destabilized by mutation to leucine. Residue 415 is solvent accessible and is on the same face of the MH2 domain as the L3 loop, such that the inhibitory activity of the p.P415L variant might also be affected (structure not shown). The p.A325T variant falls outside the structurally characterized region of the MH2 domain of the SMAD family and thus the likely effects of the mutation cannot be determined in this way. Alignment of the SMAD6 protein sequences of humans, chimpanzee, mouse, chicken, and zebrafish showed that both p.P415L and p.C484F variants occurred in highly conserved regions within the MH2 domain (Fig. 2).

### *SMAD6* Mutants Show Impaired Inhibition of BMP Signaling In Vitro

**Figure 4:**
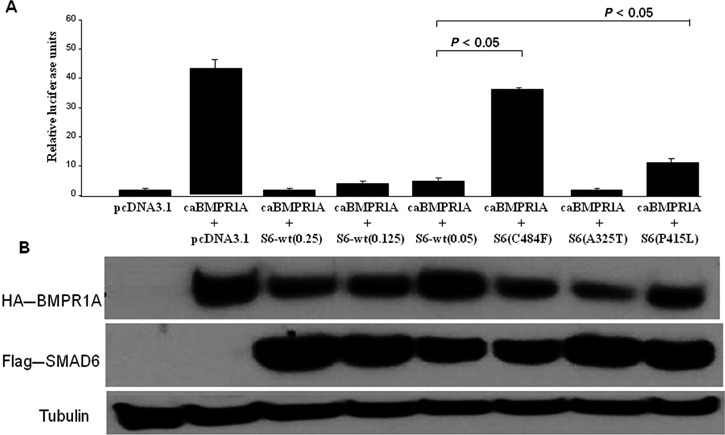
Inhibitory effects of SMAD6 variants on BMP signaling monitored via the BRE–luc transcriptional reporter. A: Firefly luciferase activity of the BRE–luc reporter induced by constitutively active BMPR1A (caBMPR1A) was measured as mean relative luciferase units ± SEM. B: Immunoblots show levels of expression of caBMPR1A and SMAD6 proteins; endogenous α-tubulin was used as a loading control. The level of wild-type (wt) SMAD6 protein was titrated by using different concentrations of plasmid in the transfections (0.25, 0.125, and 0.05 μg, as indicated in the figure). In all cases, wt SMAD6 protein led to inhibition of caBMPR1A activity. In contrast, SMAD6 (p.C485F) showed almost complete loss of inhibitory activity, SMAD6 (p.P415L) showed partial loss of activity, and there was no detectable effect of SMAD6 (p.A325T).

The MH2 domain is a critical region for SMAD6 protein function, which includes the “L3 loop,” a region required for interaction with the BMP type I receptors; the SMAD6 MH2 domain alone is sufficient to inhibit type I receptor function [Hanyu et al., 2001]. The inhibitory effects of the MH2 domains of the variant SMAD6 proteins were therefore compared with that of the wt protein on BMP-responsive transcriptional activity using a BRE–luc transcriptional reporter. SMAD6 has been reported to preferentially inhibit BMPR1A signaling [Goto et al., 2007]; therefore, caBMPR1A was selected as the signal transducer in the cell system. The MH2 domain of p.A325T mutant SMAD6 functioned in the same way as the wt MH2 domain of SMAD6 in this assay and appeared to have normal SMAD6 activity. However, the MH2 domain of p.P415L and p.C484F mutant SMAD6 proteins inhibited BMP signaling less efficiently than the equivalent domain of wt SMAD6, with relative luciferase readings sevenfold (p.P415L) and 24-fold (p.C484F) higher than the wt (Fig. 4). The p.P415L mutant appeared to be hypomorphic (*P* < 0.05 for difference with wt), whereas the p.C484F mutant appeared to be a null allele (*P* < 0.05 for difference with wt) as there was almost no inhibitory effect of the p.C484F mutant SMAD6 MH2 domain on caBMPR1A activity in this assay (Fig. 4).

### SMAD6 p.C484F Shows Impaired Inhibition of BMP-Induced ALP Activity

**Figure 5:**
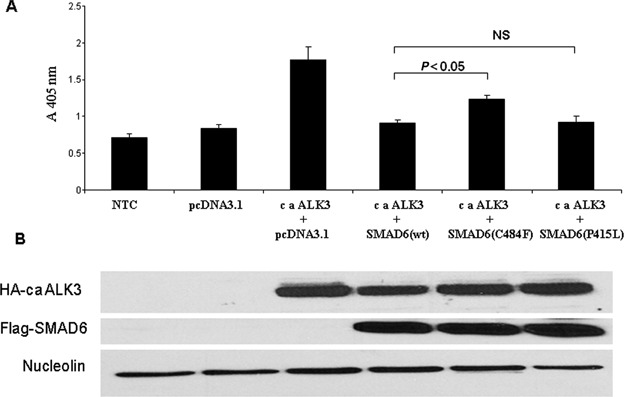
Inhibitory effects of SMAD6 variants on BMP signaling monitored via alkaline phosphatase (ALP) activity. A: ALP activity of constitutively active BMPR1A (caBMPR1A)-transfected C2C12 cells with and without SMAD6c variants was measured as absorbance at 405 nm per mg/ml protein. B: Immunoblots show levels of expression of caBMPR1A and SMAD6 protein; endogenous nucleolin was used as a loading control. SMAD6 (pC485F) showed significant loss of inhibitory activity, whereas there was no detectable effect of SMAD6 (pP415L).

To determine whether the MH2 domains of mutant SMAD6 proteins also had a less marked inhibitory effect on osteogenic potential than wt, C2C12 cells were transiently cotransfected with caBMPR1A and either wt SMAD6, p.P415L, or p.C484F expression constructs. Osteogenic potential was assessed by induction of ALP activity, a commonly used early marker of osteoblast differentiation. Quantitative analysis revealed that the p.C484F mutant showed reduced capacity to inhibit ALP activity (*P* < 0.05; Fig. 5), suggesting that mutant protein had lower efficacy in preventing tissue calcification. The p.P415L mutant, which was suggested by homology modeling and luciferase assay to be less deleterious to SMAD6 function than the p.C484F mutant, inhibited ALP activity to the same extent as the wt SMAD6C protein.

## Discussion

This is the first description of a human disease associated with mutations in *SMAD6*, and it suggests that genetic variants in the MH2 domain of *SMAD6* contribute to increased risk of CVM. In a cohort of 436 CVM patients, we found three heterozygous nonsynonymous variants in the MH2 domain of *SMAD6* that were absent in 1,000 Caucasian controls of British ancestry free of CVM [Palomino-Doza et al., 2008]. Furthermore, no nonsynonymous variants in the *SMAD6* MH2 domain were detected in two recent high-throughput sequencing projects involving a total of 829 individuals [Durbin et al., 2010; Li et al., 2010].

Two of the nonsynonymous variants in SMAD6 protein (p.C484F and p.P415L) led to amino acid changes in evolutionarily conserved residues and were predicted to affect the protein structure. Furthermore, both mutations were clearly deleterious to function in BMP signaling assays. The patient heterozygous for the p.C484F *SMAD6* variant allele was found to have a bicuspid aortic valve with mild aortic stenosis and aortic coarctation at the age of 30 years in the course of investigation for hypertension, and the coarctation was repaired. He subsequently developed significant aortic stenosis and underwent aortic valve replacement and rerepair of the aortic arch. At his second operation, it was noted that the transverse aortic arch, proximal to and distant from the previous conduit, was heavily calcified. It is possible that this is a consequence of the reduced efficiency of the p.C484F mutant SMAD6 in inhibiting osteogenic potential (Fig. 5). There was no evidence of inappropriate calcification in noncardiovascular tissues.

The other functionally significant SMAD6 variant we discovered (p.P415L) was present in a patient who presented with a heart murmur at 18 months and was found to have a bicuspid aortic valve with moderate aortic stenosis. There was no evidence of coarctation. Both patients carrying functionally significant *SMAD6* variants had bicuspid aortic valves, the commonest cardiovascular malformation, occurring in approximately 1% of the adult population. There is a phenotypic spectrum in this condition dependent on the degree of valvular malformation, ranging from severe aortic stenosis in the neonatal period to the usual presentation either as an asymptomatic murmur or established aortic stenosis in adult life. As this cohort of CVM cases was mainly recruited through a pediatric cardiology service, the numbers with bicuspid aortic valve were relatively small (24/436). It will be interesting to test whether *SMAD6* mutations are over-represented in a larger cohort of CVM patients with bicuspid aortic valves and other aortic malformations.

In the *Smad6* knockout mouse originally described by Galvin et al. (2000), multiple cardiovascular developmental abnormalities, including hyperplastic thickening of the cardiac valves and aortic ossification, are present. The association of *SMAD6* mutations with an aortic stenosis phenotype in the patients described in this study is entirely consistent with those observations and with a similar role for SMAD6 in human cardiovascular development. Our findings are also consistent with the expression of SMAD6 in the cardiac valves and outflow tract, which continues into adult life in mouse [Galvin et al., 2000], and with a recent clinical phenotypic study showing that reduced SMAD6 expression was associated with calcification of the aortic valve [Ankeny et al., 2011].

Exon-focused sequencing of two other genes (*BMPR2* and *BMPR1A*) in the BMP signaling pathway revealed no nonsynonymous variants. We therefore conclude that such variants are uncommon in these genes in CVM patients; however, sequencing of much larger number of cases would be required to exclude prevalences of 1–2%. On the basis of information from mouse models, there is a significant involvement of other BMP- and transforming growth factor β (TGFβ)-related genes in cardiac development that also warrants further investigation in congenital heart disease [Arthur and Bamforth, 2011; Wang et al., 2011]. Variants in some of these genes have already been shown to be associated with cardiovascular abnormalities in human studies, for example, *Nodal*, *GDF1*, *TGFβ3*, and *BMPR2* [Beffagna et al., 2005; Karkera et al., 2007; Mohapatra et al., 2009; Roberts et al., 2004; Roessler et al., 2009]. In one interesting case, a dominant-negative form of the BMP receptor ALK2 was found in a patient with endocardial cushion defects [Smith et al., 2009].

Although a few families have been described in which CVM segregates in a Mendelian fashion, for example, due to mutations in cardiac transcription factors [Garg et al., 2003; Schott et al., 1998], these families are exceptional and usually the inheritance pattern is less obvious; indeed, in the majority of cases, there is a single affected individual in a family. On analysis of pedigrees, however, the risk is clearly increased in the relatives of affected individuals, indicating a significant genetic contribution [Burn et al., 1998; Oyen et al., 2009]. These recurrence risks are compatible with the presence of multiple genetic risk variants of incomplete penetrance, likely interacting with environmental factors. We, and others, have already shown that incompletely penetrant alleles in key genes can predispose to CVM [Goldmuntz et al., 2001; Griffin et al., 2010; McElhinney et al., 2003; Sperling et al., 2005]. Future studies utilizing the rapidly increasing power of genome sequencing technologies to interrogate a much wider range of candidate genes, and eventually the whole exome, for rare variants that predispose to CVM will be of great interest. In the context of SMAD6, our results clearly demonstrate statistically significant differences between some of the mutant and wt SMAD6 proteins with respect to inhibitory activity in the in vitro assays, but the quantitative effects of different levels of mutant constructs remain to be explored, as do the downstream effects of BMP ligand treatments in the mutant cells in vivo. Nevertheless, results such as we describe here could have important implications for clinical practice. If a number of genes with contributions of similar magnitude as *SMAD6* to the risk of aortic abnormalities could be identified, screening of such genes to provide individual-specific counseling about recurrence risks to offspring would be a valuable addition to the current practice.
