# Supplementary material for: Nonsynonymous variants in the SMAD6 gene predispose to congenital cardiovascular malformation
Source: Hum Mutat. 2012 Jan 20;33(4):720–7. doi: 10.1002/humu.22030 (PMC3492913; doi:10.1002/humu.22030)
Supplement: Supplementary file 1 [file humu0033-0720-SD1.pdf]

**Supp. Table S1. Cardiac phenotypes of CVM patients**

| **Cardiac phenotype** | **Patient Group 1*** | **Non-synonymous variants** | **Patient Group 2*** | **Non-synonymous variants** | **Total** |
|---|---|---|---|---|---|
| Aortic stenosis | 4 | | 19 | | **23** |
| Aortic stenosis with bicuspid aortic valve | 4 | | 6 | [SMAD6 p.P415L] | **10** |
| Atrial septal defect | 29 | | 50 | | **79** |
| Atrioventricular septal defect | 8 | | 21 | | **29** |
| Bicuspid aortic valve | 0 | | 3 | | **3** |
| Coarctation of aorta | 3 | | 23 | | **26** |
| Coarctation of aorta with bicuspid aortic valve | 4 | [SMAD6 p.C484F]# | 7 | | **11** |
| Double inlet left ventricle | 4 | | 11 | | **15** |
| Ebstein's anomaly | 3 | | 6 | | **9** |
| Hypoplastic left heart | 2 | | 8 | | **10** |
| Isomerism | 2 | | 8 | | **10** |
| Mitral atresia | 0 | | 2 | | **2** |
| Mitral valve regurgitation | 0 | | 3 | [SMAD6: p.A325T] | **3** |
| Patent ductus arteriosus | 3 | | 15 | | **18** |
| Patent foramen ovale | 4 | | 6 | | **10** |
| Pulmonary atresia | 0 | | 4 | | **4** |
| Pulmonary atresia + ventricular septal defect | 0 | | 10 | | **10** |
| Pulmonary stenosis | 2 | | 3 | | **5** |
| Tetralogy of Fallot | 0 | | 7 | | **7** |
| Transposition of great arteries | 4 | | 52 | | **56** |
| Transposition of great arteries-corrected | 0 | | 9 | | **9** |
| Tricuspid atresia | 4 | | 12 | | **16** |
| Truncus arteriosus | 0 | | 9 | | **9** |
| Ventricular septal defect | 10 | | 40 | | **50** |
| Other | 0 | | 12 | | **12** |
| **Total** | **90** | | **346** | | **436** |

Numbers of patient with different cardiac phenotypes in each group are listed.
*The entire coding sequences of *BMPR2* (NM_001204.6), *BMPR1A* (NM_004329.2) and *SMAD6* (NM_005585.4) genes were sequenced in the first patient group, whilst only the coding region for the MH2 domain of *SMAD6* was sequenced in the second patient group.
# This patient also had aortic stenosis.
